# Effect of oilseed type on milk fatty acid composition of individual cows, and also bulk tank milk fatty acid composition from commercial farms

**DOI:** 10.1017/S1751731116001403

**Published:** 2016-07-08

**Authors:** K. E. Kliem, D. J. Humphries, C. K. Reynolds, R. Morgan, D. I. Givens

**Affiliations:** School of Agriculture, Policy and Development, Sustainable Agriculture and Food Systems Division, University of Reading, Earley Gate, Reading, Berkshire RG6 6AR, UK

**Keywords:** milk fatty acids, saturated fatty acids, linseed, rapeseed

## Abstract

Supplementing dairy cow diets with oilseed preparations has been shown to replace milk saturated fatty acids (SFA) with mono- and/or polyunsaturated fatty acids (MUFA, PUFA), which may reduce risk factors associated with cardio-metabolic diseases in humans consuming milk and dairy products. Previous studies demonstrating this are largely detailed, highly controlled experiments involving small numbers of animals, but in order to transfer this feeding strategy to commercial situations further studies are required involving whole herds varying in management practices. In experiment 1, three oilseed supplements (extruded linseed (EL), calcium salts of palm and linseed oil (CPLO) and milled rapeseed (MR)) were included in grass silage-based diets formulated to provide cows with ~350 g oil/day, and compared with a negative control (Control) diet containing no supplemental fat, and a positive control diet containing 350 g/cow per day oil as calcium salt of palm oil distillate (CPO). Diets were fed for 28-day periods in a 5×4 Latin Square design, and milk production, composition and fatty acid (FA) profile were analysed at the end of each period. Compared with Control, all lipid supplemented diets decreased milk fat SFA concentration by an average of 3.5 g/100 g FA, by replacement with both *cis*- and *trans*-MUFA/PUFA. Compared with CPO, only CPLO and MR resulted in lower milk SFA concentrations. In experiment 2, 24 commercial dairy farms (average herd size±SEM 191±19.3) from the south west of the United Kingdom were recruited and for a 1 month period asked to supplement their herd diets with either CPO, EL, CPLO or MR at the same inclusion level as the first study. Bulk tank milk was analysed weekly to determine FA concentration by Fourier Transform mid-IR spectroscopy prediction. After 4 weeks, EL, CPLO and MR all decreased herd milk SFA and increased MUFA to a similar extent (average −3.4 and +2.4 g/100 g FA, respectively) when compared with CPO. Differing responses observed between experiments 1 and 2 may be due in part to variations in farm management conditions (including basal diet) in experiment 2. This study demonstrates the importance of applying experimental research into commercial practice where variations in background conditions can augment different effects to those obtained under controlled conditions.

## Implications

Saturated fatty acids (SFA) are thought to increase the risk of cardiovascular disease and in many Western diets, milk/dairy products are a major source of SFA. Supplementing dairy cow diets with oilseeds decreases milk SFA content, but to date this has not been demonstrated under both experimental and commercial conditions. This study showed that at low levels, oilseed supplements can have meaningful effects on milk fatty acid (FA) profiles (without affecting cow performance) both experimentally and commercially. Therefore, these oilseed preparations could all be used in dairy cow feeding as a part of an overall strategy for replacing SFA in the human diet.

## Introduction

There is evidence that cardiovascular disease risk can be reduced by the isoenergetic replacement of SFA with *cis*-monounsaturated fatty acids (MUFA) or polyunsaturated fatty acids (PUFA) in the human diet (Vafeiadou *et al*., 2015). In the United Kingdom, milk and dairy products contribute about 25% and 28% of total SFA consumed by men and women, respectively (Bates *et al*., 2014), with higher contributions in other countries (Hulshof *et al*., 1999). However, instead of advocating population-wide decreases in milk and dairy product consumption, altering the FA composition of milk and dairy products by replacing SFA with MUFA and/or PUFA offers an opportunity to lower SFA intake while maintaining the contribution of these foods to the balanced human diet.

Supplementing dairy cow diets with oilseed preparations is an effective means of replacing milk fat SFA with unsaturated FA (Glasser *et al*., 2008). Kliem *et al*. (2011) reported a reduction in SFA from 67 to 57 g/100 g FA, whilst increasing *cis*-MUFA from 25 to 33 g/100 g FA after cows consumed around 1.2 kg oil as milled high oleic acid rapeseed/day. A study involving extruded linseed (EL) reported a change in SFA from 75 to 57 g/100 g FA, with SFA being replaced by MUFA (20 to 34 g/100 g FA) and PUFA (2.5 to 5.7 g/100 g FA) after feeding 960 g oil from the supplement (Ferlay *et al*., 2010). However these studies involved feeding high levels of oilseeds, which may decrease milk fat and protein concentration, especially over longer periods (Lerch *et al*., 2012a). In addition, costs may restrict feeding at these levels on a herd basis.

To our knowledge there are few published studies investigating the effects of supplemental oilseeds on milk FA composition in commercial herds. Stergiadis *et al*. (2014) investigated the effects of full-fat rolled linseed or rapeseed supplementation in two groups of cows over 6-week periods, but fed 1.25 kg/cow per day of rapeseed or 1.5 kg/cow per day linseed, which may not be practical.

The objective of this study was to investigate whether selected oilseed supplements, when fed at similar oil intake, resulted in decreased SFA and increased unsaturated FA concentrations in milk fat, in both a controlled, experimental study and on commercial farms with the normal variation associated with commercial practice.

## Material and methods

### Experiment 1: individual cow study

#### Experimental design, animals and management

All experimental procedures used were licensed, regulated and inspected by the UK Home Office under the Animals (Scientific Procedures) Act, 1996. Five multiparous Holstein-Friesian cows of mean±standard error parity 3.6±0.93, milk yield 33.4±1.18 l/day and 216±8.8 days in lactation were used. Animals were randomly allocated to one of five treatments in a 5×4 Latin Square design experiment with 28-day periods. Cows were housed in a cubicle yard with rubber chip filled mattresses and wood shavings as required as additional bedding. In the cubicle yard individual feeding was achieved using an electronic identification system and pneumatic feed barrier (Insetec, Marknesse, the Netherlands). Clean water was constantly available via a trough system. Cows were milked through a conventional herringbone parlour twice daily at 0600 and 1600 h.

#### Experimental diets

Diets were offered as total mixed rations (TMR; forage : concentrate ratio 50 : 50 on a dry matter (DM) basis) with the forage consisting of maize silage and grass silage (250 and 750 g/kg of forage DM, respectively; Table 1). Treatments consisted of a basal diet (Control), with the concentrate portion containing a minimal amount (6.8 g/kg DM) of a commercial fat supplement (calcium salts of palm oil FA (CPO); Megalac^®^, Volac International Ltd, Royston, UK). Treatment diets were the basal diet with the addition of 350 g oil/cow per day supplied as CPO (17 g/kg DM), EL (55 g/kg DM; Lintec, BOCM Pauls Ltd, Wherstead, Suffolk, UK), calcium salts of palm and linseed oil (CPLO) FA (28 g/kg DM; CPLO; Flaxpro, Volac International Ltd), or milled rapeseed (MR, 39 g/kg DM; provided for the study by BOCM Pauls Ltd). The MR supplement was manufactured by crushing rapeseed in a hammer mill using wheat feed as a carrier in proportions of 75 : 25 on a fresh weight basis, respectively. Diets were formulated to be isonitrogenous (Table 1). Cows were offered diets as equal meals at 0830 and 1600 h.

**Table 1:** Ingredients and chemical composition of experimental diets in experiment 1 (g/kg DM or as stated)

	Treatments				
	Control	CPO	EL	CPLO	MR
Ingredients					
Maize silage	120	120	120	120	120
Grass silage	360	360	360	360	360
Grass hay	15	15	15	15	15
Straw	15	15	15	15	15
Concentrate mix^1^	416	416	416	416	416
Soya bean meal	9	16	0	8	4
Soya bean hulls	55	31	9	29	22
Sodium bicarbonate	4	4	4	4	4
Salt^2^	4	4	4	4	4
Limestone	2	2	2	2	2
CPO	0	17	0	0	0
EL	0	0	55	0	0
CPLO	0	0	0	28	0
MR	0	0	0	0	39
Chemical composition					
DM (g/kg fresh)	597	599	598	598	598
Organic matter	884	881	884	884	884
CP	185	186	185	189	189
NDF	348	333	328	339	338
ADF	207	195	191	199	198
Starch	133	133	136	136	137
Water soluble carbohydrates	25.4	26.9	26.1	25.8	25.8
ME (MJ/kg DM)	11.8	12.0	12.1	11.7	11.7
Fatty acids					
16:0	6.6	13.7	7.1	9.7	8.0
18:0	0.78	1.37	1.15	1.32	1.04
18:1 *cis*-9	6.1	11.5	7.9	12.3	13.7
18:2 n-6	8.6	10.2	9.3	10.6	11.2
18:3 n-3	4.4	4.4	6.6	7.9	5.4
Total fatty acids	32.6	47.3	38.0	48.0	45.9

CPO=calcium salts of palm oil distillate; EL=extruded linseed; CPLO=calcium salts of palm and linseed oil distillate; MR=milled rapeseed; DM=dry matter; ME=metabolisable energy; DDGS=dried distillers grains with solubles.

1 Containing (g/kg DM): cracked wheat, 102; DDGS wheat, 43; soya bean meal 67; rapeseed meal, 73; palm kernel meal, 32; molassed sugar beet feed, 32; soya hulls, 30; Megalac^®^, 7; molasses, 17; urea, 5; minerals (KW Alternative Feeds Ltd, Barrow Hill Barns, Andover, UK), 9.

2 Dairy Direct, Church Farm, Bury St Edmunds, UK.

#### Experimental sampling

Individual forage components of experimental diets and the TMR were sampled daily and added to a weekly composite sample. Oven DM contents were determined daily by drying at 100°C for 23 h to ensure that the DM composition of experimental diets was maintained. Concentrate components were sampled once per week. Refused feed was removed before the morning feed and weighed daily; fresh weights were recorded and during measurement weeks a weekly composite of refused feed was dried at 60°C for 48 h to determine individual daily dry matter intakes (DMIs). Samples of dietary components, TMR and refusals (if appreciable) were retained at −20°C for chemical analysis.

Milk yield was recorded daily throughout the study. Samples of milk for the determination of fat, protein and lactose were collected at each milking during the last 7 days of each period and preserved with potassium dichromate (1 mg/ml, Lactabs; Thompson and Capper, Runcorn, UK). Additional samples of unpreserved milk were collected during the last 24 h of each experimental period, stored at −20°C until composited according to milk yield, and used for FA analysis.

#### Chemical analysis

Chemical composition of oven dried (60°C), milled (1 mm screen) samples of forages, concentrates and TMR were determined using methods outlined by (Kliem *et al*., 2008) for NDF, CP, water soluble carbohydrates, ether extract, starch, metabolisable energy and FA composition (subsamples of TMR). Feed FA quantification was achieved using methyl heneicosanoate (H3265; Sigma-Aldrich Company Ltd, Dorset, UK) in toluene (1 mg/ml) as an internal standard.

Milk fat, CP and lactose were determined by mid-IR spectroscopy (Foss Electric Ltd, York, UK). Lipid in 1 ml milk was extracted, transesterified and the resulting fatty acid methyl esters separated using the methods of Kliem *et al*., (2008). All milk FA results were expressed as g/100 g total FA.

#### Data analysis

Intake, milk production, milk composition and milk FA composition data were averaged for each cow and analysed using the mixed procedure of Statistical Analysis Systems (SASsoftware package version 8.2; SAS Institute, Cary, NC, USA), testing fixed effects of period and treatment and random effect of cow, with period as a repeated effect within cow. Compound symmetry, heterogeneous compound symmetry, first-order autoregressive or a heterogeneous first-order regressive covariance structure were used for repeated measures analysis, based on goodness-of-fit criteria for each variable analysed. Where treatment was significant, treatment means were compared using the difference procedure of the least squares means option. Least square means±SEM were reported and treatment effects were considered significant at *P*<0.05.

### Experiment 2: commercial farm study

#### Recruitment of farms and experimental design

A total of 24 farms from the south west of the UK were enrolled in the study. Farms registering interest were selected via cross referencing with feed suppliers and the milk testing laboratory. All recruited farms were planning on feeding a lipid supplement to their whole herds during the winter of 2011 to 2012. Two farms dropped out due to feeding system incompatibility. Background data from each farm was obtained before the study (Table 2), and recruited herds were composed of either Holstein (*n*=20) or Holstein-Friesian (*n*=2) cattle. Farms were allocated to one of four treatment groups (CPO, EL, CPLO and MR), and during the winter period fed these supplements (supplied by the same manufacturers as in experiment 1) at their chosen feeding rate. For a 4-week period farms were requested to adjust the supplemental feeding rate to provide each cow with 350 g oil/day. The treatment period start date varied from farm to farm, depending on supplement deliveries and other farm management considerations (CPO start dates ranged from 10 December 2011 to 27 February 2012; EL ranged from 30 January 2012 to 17 February 2012; CPLO ranged from 1 January 2012 to 12 March 2012; MR ranged from 7 February 2012 to 22 February 2012). The volume of milk sold from each farm during the treatment period was obtained and used together with number of cows in milk before the treatment period, to calculate average yield per cow.

**Table 2:** Summary of commercial farms enrolled onto experiment 2

	Diet group				
	CPO	EL	CPLO	MR	SEM^1^
No. of farms	5	6	6	5	–
Average herd size^2^	253	152	213	153	19.3
Average number of cows in milk^2^	226	119	180	141	17.8
Annual yield (l/cow)^2^	8870	8607	8044	8313	267.7
Average grass silage : maize silage in forage portion of diet (fresh weight basis)^2^	57 : 43	75 : 25	55 : 45	59 : 41	–
Ratio of all-year-round : seasonal calving herds^2^	2 : 3	5 : 1	2 : 4	4 : 1	–

CPO=calcium salts of palm oil distillate; EL=extruded linseed; CPLO=calcium salts of palm and linseed oil distillate; MR=milled rapeseed.

1 SEM for *n*=22 values.

2 Information obtained from National Milk Records or from farms directly before the study.

#### Experimental sampling

During the winter period (7 November 2011 to 23 April 2012) bulk milk was sampled at every collection, and preserved with potassium dichromate preservative (1 mg/ml, Lactabs) before analysis. Immediately preceding and at 1 week periods following instigation of the treatment period (until the end of week 4, so *n*=5 samples in total) bulk tank samples were sampled as above.

#### Chemical analysis

Milk fat, CP, lactose, total SFA, total MUFA and total PUFA were determined in all samples by mid-IR spectroscopy. The results for FA groups (as g/100 g milk) were converted to g/100 g milk FA, by first calculating total FA (milk fat as g/100 g milk multiplied by 0.95; International Dairy Federation (IDF), 2010). A sample of each supplement was analysed for FA profile using the methods outlined above.

#### Data analysis

Calculated milk yield from the beginning and end of experimental period was analysed using a GLM in Minitab (Minitab^®^ 16) that included effects of treatment group, time (beginning and end) and their interaction. Milk compositional data and major FA group data for each week of the treatment period were analysed using the mixed procedure of SAS, testing fixed effects of treatment, week and treatment by week interactions and random effect of farm, with week as a repeated effect within farm. Values from each farm at week 0 and the amount of supplemental lipid fed daily to each cow at week −1 were included as covariates. Compound symmetry, heterogeneous compound symmetry, first-order autoregressive or a heterogeneous first-order regressive covariance structure were used for repeated measures analysis, based on goodness-of-fit criteria for each variable analysed. Least square means for each treatment and week were compared. Treatment effects were considered significant at *P*<0.05.

## Results

### Experiment 1: individual cow study

Overall diets were readily consumed with cows maintaining satisfactory DMIs (24.0 kg DM/day) and milk yields (30.6 kg/day) throughout the study. Chemical analysis of the five treatment diets showed little difference in most components (Table 1). FA analysis of TMR subsamples demonstrated that the CPO diet was highest in 16:0 and 18:0, MR diet highest in *cis*-9 18:1 and 18:2 n-6 and CPLO diet highest in 18:3 n-3 (Table 1). Overall, there was little difference between the total FA content of CPO, CPLO and MR, whereas that of EL (and Control) was lower (Table 1).

There was no effect of treatment on DMI, milk yield or milk composition (Table 3), but an effect of diet (*P*<0.001) was observed for FA intake. Cows fed CPO consumed the highest (*P*<0.05) amounts of 16:0 and 18:0, and those fed MR consumed greater (*P*<0.05) quantities of *cis*-9 18:1. The greatest (*P*<0.05) amount of 18:2 n-6 and 18:3 n-3 was consumed by cows fed CPLO (Table 3).

**Table 3:** Effect of lipid supplement on dry matter and fatty acid intake, and milk and constituent yield in experiment 1 (least square mean results)

	Treatments^1^						*P* ^2^	
	Control	CPO	EL	CPLO	MR	SEM^3^	Period	Diet
DM intake (kg/day)	24.1	23.6	24.1	24.1	24.2	0.79	0.005	0.870
Fatty acid intake (g/day)								
16:0	157^d^	320^a^	172^d^	234^b^	194^c^	8.6	0.027	<0.001
18:0	18.7^d^	32.0^a^	27.8^b^	31.9^a^	25.3^c^	0.95	0.012	<0.001
18:1 *cis*-9	145^e^	270^c^	191^d^	297^b^	330^a^	9.0	0.011	<0.001
18:2n-6	208^d^	239^c^	224^c^	355^a^	271^b^	8.0	0.005	<0.001
18:3n-3	106^d^	104^d^	160^b^	192^a^	130^c^	4.5	0.008	<0.001
Total fatty acids	781^c^	1112^a^	917^b^	1159^a^	1111^a^	35.0	0.007	<0.001
Yield								
Milk (kg/day)	30.1	30.2	30.5	31.5	30.6	1.20	0.470	0.770
Fat (g/day)	1104	1161	1130	1161	1133	71.9	0.257	0.569
Protein (g/day)	962	949	970	1004	968	37.6	0.050	0.454
Lactose (g/day)	1354	1347	1377	1425	1382	58.5	0.364	0.734
Concentration (g/kg)								
Fat	37.1	38.4	37.4	36.8	36.8	2.17	0.287	0.064
Protein	32.0	31.6	31.6	31.8	31.9	0.71	0.136	0.902
Lactose	44.8	44.3	45.2	45.3	45.3	0.95	0.448	0.276

DM=dry matter. ^a,b,c,d^Where there is an overall diet effect, values within rows with differing superscripts are significantly (*P*<0.05) different.

1 CPO, EL, CPLO and MR are diets containing 350 g oil/day equivalent of calcium salts of palm oil, extruded linseed, calcium salts of palm and linseed oil and milled rapeseed, respectively.

2 Refers to the significance of overall effect of period and diet.

3 SEM for *n*=20 measurements.

Diet affected milk fat short and medium chain SFA concentrations (Table 4), with CPLO decreasing (*P*<0.05) 8:0 and 10:0 concentrations. The lipid supplemented diets all decreased (*P*<0.05) 14:0 and 15:0 concentrations when compared with Control, and there were variations in concentrations of 13:0 iso, 14:0 iso and 15:0 anteiso depending on treatment diet. There was no difference in 16:0 concentration between Control and CPO, but the other treatments all lowered (*P*<0.05) 16:0 concentration compared with both Control and CPO. In contrast 18:0 was increased (*P*<0.05) when EL, CPLO and MR were fed compared with Control and CPO. This led to an overall effect of treatment diet (*P*=0.022) on total SFA, but only CPLO and MR were lower (*P*<0.05) than Control.

**Table 4:** Effect of lipid supplement on milk fatty acid composition in experiment 1 (least square mean results as g/100 g fatty acids)

	Treatments^1^						*P* ^2^	
Fatty acid	Control	CPO	EL	CPLO	MR	SEM^3^	Period	Diet
4:0	2.63	2.55	2.68	2.71	2.64	0.054	0.021	0.194
6:0	1.76	1.65	1.76	1.64	1.72	0.056	0.109	0.185
8:0	1.14^a^	1.04^ab^	1.10^a^	0.97^b^	1.13^a^	0.063	0.026	0.035
10:0	2.86^a^	2.50^ab^	2.75^a^	2.22^b^	2.63^a^	0.186	0.019	0.029
10:1 *cis*-9	0.28	0.25	0.27	0.26	0.25	0.020	<0.001	0.122
12:0	3.78	3.29	3.57	2.97	3.42	0.231	0.035	0.059
12:1 *cis*-9	0.10	0.09	0.09	0.09	0.09	0.008	0.017	0.264
13:0	0.11	0.07	0.10	0.08	0.09	0.008	0.120	0.080
13:0 iso	0.032^b^	0.030^c^	0.035^a^	0.027^d^	0.029^cd^	0.002	0.036	<0.001
13:0 anteiso	0.087	0.079	0.080	0.072	0.077	0.0063	0.005	0.410
14:0	11.4^a^	10.3^b^	10.9^ab^	10.3^b^	10.6^b^	0.40	0.003	0.015
14:0 iso	0.087^a^	0.072^c^	0.080^ab^	0.077^bc^	0.084^ab^	0.0078	0.038	0.017
14:1 *trans*-9	0.27^a^	0.23^c^	0.25^b^	0.23^c^	0.24^bc^	0.006	<0.001	0.007
14:1 *cis*-9	1.07	0.98	0.96	0.96	0.90	0.080	0.009	0.299
15:0	1.14^a^	0.90^c^	1.00^b^	0.91^bc^	0.93^bc^	0.054	0.075	0.003
15:0 anteiso	0.56^a^	0.48^c^	0.51^b^	0.45^d^	0.46^d^	0.019	0.045	<0.001
16:0	31.4^ab^	32.5^a^	28.7^bc^	27.9^c^	27.2^c^	0.65	0.594	0.022
16:0 iso	0.20	0.19	0.20	0.19	0.18	0.022	0.002	0.872
16:1 *trans*-6+7+8	0.048	0.078	0.057	0.126	0.086	0.0234	0.178	0.151
16:1 *trans*-9	0.058^b^	0.062^c^	0.058^b^	0.068^a^	0.055^b^	0.0092	0.265	0.024
16:1 *trans*-11+12+13	0.18	0.19	0.18	0.19	0.18	0.008	0.868	0.101
16:1 *cis*-9^4^	1.32	1.33	1.15	1.15	1.06	0.095	0.098	0.117
16:1 *cis*-11	0.54^a^	0.49^b^	0.49^b^	0.47^b^	0.48^b^	0.029	0.025	0.024
16:1 *cis*-13	0.21^a^	0.17^cd^	0.18^bc^	0.16^d^	0.19^ab^	0.018	0.206	0.009
17:0	0.54^a^	0.47^bc^	0.49^b^	0.46^c^	0.48^bc^	0.018	0.002	<0.001
17:0 iso	0.36^a^	0.33^b^	0.34^ab^	0.34^b^	0.34^b^	0.018	0.002	0.032
17:1 *cis*-9	0.16	0.16	0.16	0.14	0.15	0.012	0.779	0.481
18:0	9.76^c^	8.91^d^	11.19^b^	11.69^b^	13.20^a^	0.42	0.008	0.007
18:0 iso	0.046	0.045	0.037	0.038	0.037	0.0052	0.458	0.105
18:1 *trans* total	2.84^c^	3.11^bc^	3.30^bc^	4.07^a^	3.30^b^	0.153	0.221	0.006
18:1 *cis* total	20.1	22.2	21.9	23.2	22.9	0.75	0.098	0.066
Non-CLA 18:2 total^5^	2.94^bc^	2.94^c^	3.26^a^	3.17^ab^	2.85^c^	0.155	0.107	0.006
CLA total	0.58^bc^	0.64^b^	0.59^b^	0.73^a^	0.54^c^	0.052	0.210	0.028
18:3 n-6	0.032^a^	0.029^a^	0.029^a^	0.023^b^	0.023^b^	0.0057	0.398	0.012
18:3 n-3	0.59^c^	0.52^c^	0.92^a^	0.80^b^	0.59^c^	0.044	0.116	<0.001
19:0^6^	0.035^c^	0.049^a^	0.043^ab^	0.047^ab^	0.042^bc^	0.0053	0.024	0.011
20:0	0.15^b^	0.14^d^	0.15^c^	0.15^b^	0.20^a^	0.007	<0.001	<0.001
20:1 *cis*-11	0.052^c^	0.058^b^	0.056^bc^	0.059^b^	0.071^a^	0.0019	0.030	0.002
20:2 n-6	0.033	0.028	0.028	0.027	0.024	0.0023	0.011	0.077
20:3 n-3	0.020	0.009	0.014	0.011	0.057	0.0058	0.087	0.059
20:4 n-6	0.15	0.14	0.13	0.12	0.15	0.008	0.631	0.152
20:5 n-3	0.053^b^	0.044^c^	0.065^a^	0.054^b^	0.047^bc^	0.0037	0.404	<0.001
22:0	0.14	0.14	0.12	0.11	0.13	0.017	0.345	0.126
22:4 n-6	0.033	0.034	0.034	0.030	0.034	0.0037	0.502	0.837
22:5 n-3	0.089^a^	0.078^b^	0.084^ab^	0.081^b^	0.084^ab^	0.0067	0.524	0.049
Σ SFA	68.0^a^	66.3^ab^	65.6^ab^	62.8^c^	65.1^bc^	0.90	0.073	0.021
Σ SFA ⩽14:0	23.7	21.8	24.0	20.8	22.4	0.86	0.060	0.215
Σ *trans* total	4.11^d^	4.37^cd^	4.84^b^	5.68^a^	4.66^bc^	0.172	0.080	<0.001
Σ *trans* MUFA	3.40^c^	3.67^bc^	3.87^ab^	4.72^a^	3.91^b^	0.154	0.098	0.003
Σ *cis* MUFA	23.8^b^	25.1^ab^	25.0^ab^	27.2^a^	26.3^a^	0.73	0.137	0.046
Σ n-6 PUFA	2.53^a^	2.57^a^	2.60^a^	2.49^a^	2.37^b^	0.16	0.113	0.012
Σ n-3 PUFA	0.91^c^	0.81^c^	1.37^a^	1.22^b^	0.90^c^	0.048	0.141	<0.001
n-6 : n-3	2.71^b^	3.12^a^	2.02^c^	2.06^c^	2.59^b^	0.075	0.609	<0.001

SFA=saturated fatty acids; MUFA=monounsaturated fatty acids; PUFA=polyunsaturated fatty acids. ^a,b,c,d^Where there is an overall diet effect, values within rows with differing superscripts are significantly (*P*<0.05) different.

1 CPO, EL, CPLO and MR are diets containing 350 g oil/day equivalent of calcium salts of palm oil, extruded linseed, calcium salts of palm and linseed oil and milled rapeseed, respectively.

2 Refers to the significance of overall effect of period and diet.

3 SEM for *n*=20 measurements.

4 Co-elutes with 17:0 anteiso.

5 All 18:2 isomers excluding CLA.

6 Co-elutes with *cis*-15 18:1.

CPLO and MR increased (*P*<0.05) total *cis*-MUFA compared with Control (Table 4), mainly due to numerical increases in *cis*-9 18:1 (Table 5). In addition, total *trans* MUFA concentrations were greater (*P*<0.05) with EL, CPLO and MR compared with Control. This was mainly due to enhanced concentrations of some of the *trans*-18:1 isomers, notably *trans*-4 18:1, *trans*-6-8 18:1, *trans*-9 18:1, *trans*-12 18:1 and *trans*-16 18:1 (Table 5).

**Table 5:** Effect of lipid supplement on milk fat 18:1 isomer composition in experiment 1 (least square mean results as g/100 g fatty acids)

	Treatments^1^						*P* ^2^	
Fatty acid	Control	CPO	EL	CPLO	MR	SEM^3^	Period	Diet
*trans*-4 18:1	0.003^d^	0.026^b^	0.018^c^	0.041^a^	0.034^ab^	0.0038	0.052	<0.001
*trans*-5 18:1	0.050	0.055	0.068	0.018	0.098	0.0505	0.572	0.584
*trans*-6-8 18:1	0.28^d^	0.33^c^	0.32^c^	0.44^a^	0.40^b^	0.015	0.020	<0.001
*trans*-9 18:1	0.21^d^	0.26^c^	0.30^b^	0.34^a^	0.30^b^	0.017	0.235	<0.001
*trans*-10 18:1	0.48	0.47	0.45	0.58	0.54	0.074	0.211	0.082
*trans*-11 18:1	0.99	1.00	0.97	1.27	1.09	0.09	0.049	0.186
*trans*-12 18:1	0.40^c^	0.44^bc^	0.52^a^	0.56^a^	0.50^ab^	0.046	0.599	0.006
*trans*-15 18:1	0.37	0.34	0.52	0.36	0.34	0.038	0.386	0.265
*trans*-16 18:1^4^	0.37^c^	0.37^c^	0.60^a^	0.56^a^	0.45^b^	0.044	0.948	<0.001
*cis*-9 18:1^5^	18.8	20.9	20.4	21.7	21.5	0.73	0.084	0.070
*cis*-11 18:1	0.61	0.60	0.59	0.60	0.71	0.034	0.597	0.067
*cis*-12 18:1	0.25^b^	0.26^b^	0.40^a^	0.37^a^	0.30^b^	0.031	0.606	0.005
*cis*-13 18:1	0.09	0.07	0.12	0.11	0.09	0.016	0.425	0.154
*cis*-16 18:1	0.057^c^	0.069^b^	0.089^a^	0.089^a^	0.072^b^	0.0053	0.174	<0.003

^a,b,c,d^Where there is an overall diet effect, values within rows with differing superscripts are significantly (*P*<0.05) different.

1 CPO, EL, CPLO and MR are diets containing 350 g oil/day equivalent of calcium salts of palm oil, extruded linseed, calcium salts of palm and linseed oil and milled rapeseed, respectively.

2 Refers to the significance of overall effect of period and diet.

3 SEM for *n*=20 measurements.

4 Co-elutes with 18:1 *cis*-14.

5 Co-elutes with 18:1 *trans-*13/14.

Treatment diet also affected milk fat PUFA concentration. Total n-6 PUFA concentration was lower (*P*<0.05) with MR compared with the other diets, mainly due to a lower concentration of *cis*-9, *cis*-12 18:2 (Table 6). There were also differences in concentrations of minor n-6 PUFA, with EL enhancing (*P*<0.05) *cis*-9, *trans*-12 and *trans*-9, *trans*-12 18:2 (Table 6) compared with the other diets. Concentrations of total n-3 PUFA were greater (*P*<0.05) with EL and CPLO compared with Control, CPO and MR (Table 4), largely due to 18:3 n-3 concentration, but also 20:5 n-3 (Table 4) and *trans*-11, *cis*-15 18:2 concentrations (Table 6). The greatest (*P*<0.05) total CLA concentration was observed after CPLO was fed (Table 4) when compared with the other treatment diets.

**Table 6:** Effect of lipid supplement on milk fat 18:2 isomer composition in experiment 1 (least square mean results as mg/100 g fatty acids)

	Treatments^1^						*P* ^2^	
Fatty acid	Control	CPO	EL	CPLO	MR	SEM^3^	Period	Diet
*trans*-11, *trans*-15 18:2	74.4^bc^	66.8^c^	94.0^a^	81.1^b^	78.6^bc^	6.41	0.007	0.010
*trans*-9, *trans-*12 18:2	2.36^b^	0.00^b^	12.55^a^	2.36^b^	2.73^b^	1.96	0.095	0.010
*cis*-9, *trans*-12 18:2	20.7^c^	26.7^bc^	45.0^a^	34.1^b^	28.5^bc^	3.49	0.013	0.004
*cis*-9, *trans*-13 18:2	201^b^	194^b^	301^a^	296^a^	230^b^	33.1	0.199	0.009
*cis-*9, *trans*-14 18:2	84.0^b^	84.9^b^	136.3^a^	112.3^a^	65.0^b^	13.6	0.675	0.008
*cis*-10, *trans*-14 18:2	143	130	129	130	131	17.1	0.877	0.975
*trans*-9, *cis*-12 18:2	29.8	29.9	40.9	40.2	29.2	2.11	0.578	0.212
*trans*-11, *cis*-15 18:2	101^b^	106^b^	191^a^	203^a^	116^b^	13.3	0.612	<0.001
*cis*-9, *cis*-12 18:2	2231^ab^	2344^a^	2240^ab^	2164^bc^	2079^c^	164.2	0.010	0.026

^a,b,c^Where there is an overall diet effect, values within rows with differing superscripts are significantly (*P*<0.05) different.

1 CPO, EL, CPLO and MR are diets containing 350 g oil/day equivalent of calcium salts of palm oil, extruded linseed, calcium salts of palm and linseed oil and milled rapeseed, respectively.

2 Refers to the significance of overall effect of period and diet.

3 SEM for *n*=20 measurements.

### Experiment 2: commercial farm study

The supplements used in the commercial farm study varied in terms of their FA profile. The CPO supplement contained the most 16:0 (48 g/100 g FA) compared with CPLO, EL and MR at 19, 7 and 5 g/100 g FA, respectively. MR was highest in *cis*-9 18:1 (57 g/100 g FA), followed by CPLO, CPO and EL at 40, 36 and 17 g/100 g FA, respectively. The greatest proportion of 18:2 n-6 was in MR (20 g/100 g FA), and EL was richest in 18:3 n-3 (51 g/100 g FA) compared with CPLO, MR and CPO with 22, 10 and <0.5 g/100 g FA, respectively.

**Figure 1:**
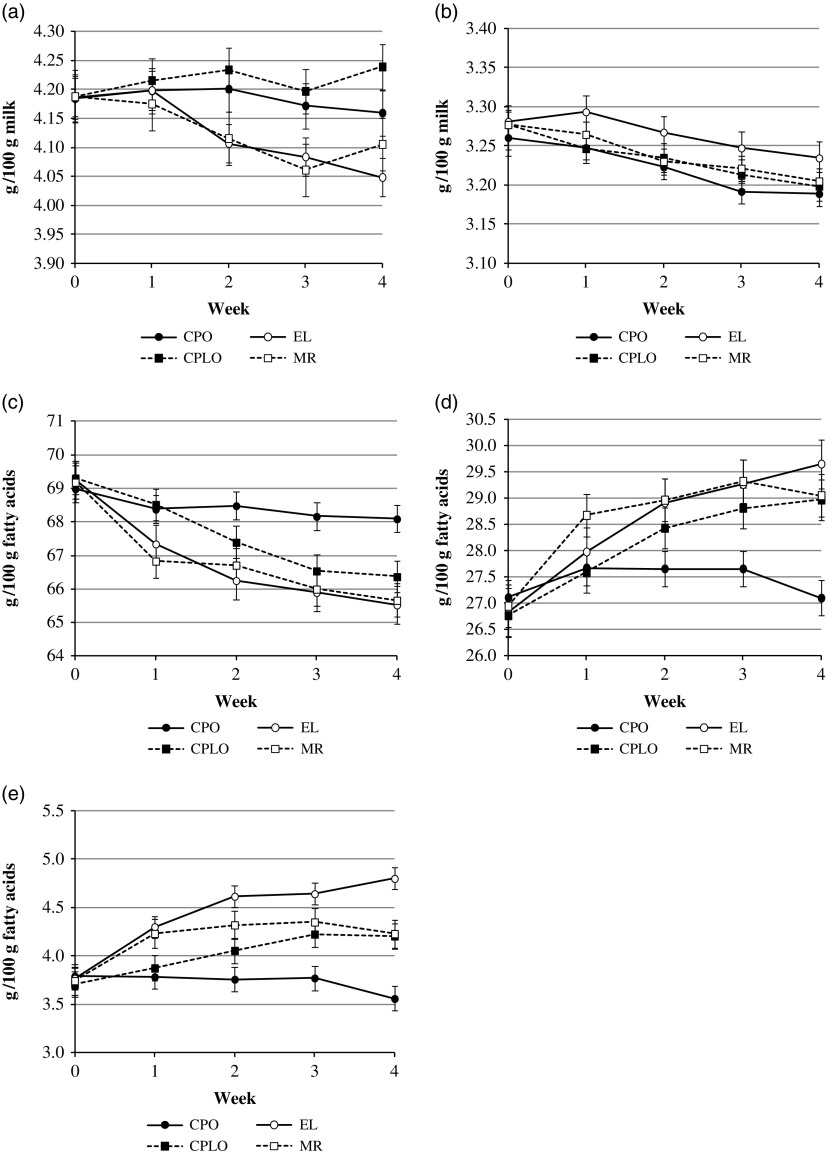
Effect of lipid supplement on (a) bulk tank milk fat (g/100 g milk), (b) bulk tank milk protein (g/100 g milk), (c) bulk tank milk saturated fatty acid concentration (g/100 g fatty acids), (d) bulk tank monounsaturated fatty acid concentration (g/100 g fatty acids) and (e) bulk tank milk polyunsaturated fatty acid concentration (g/100 g fatty acids) of commercial dairy herds over a 4-week period (experiment 2). CPO, EL, CPLO and MR are diets containing 350 g oil/day equivalent of calcium salts of palm oil, extruded linseed, calcium salts of palm and linseed oil and milled rapeseed, respectively.

There was no effect (*P*>0.05) of treatment group or time on average yield sold from the farms per cow, with yields (±SEM) for the beginning and end of the experimental periods of; CPO 30.8 *v*. 31.4 (±3.45) l/cow, EL 28.5 *v*. 29.9 (±2.44) l/cow, CPLO 26.5 *v*. 27.1 (±2.67) l/cow and MR 23.5 *v*. 24.4 (±3.00) l/cow. Over the 4-week experimental period there was no overall diet effect on milk fat (*P*=0.071) and protein (*P*=0.122) concentrations (Figure 1a and b). There was, however, an effect of week for fat (*P*=0.033) and protein (*P*<0.001). There was an overall effect (*P*=0.010) of diet on total SFA (Figure 1c). There was no difference (*P*=0.149) between weeks 4 and 0 for CPO, but EL, CPLO and MR all decreased (*P*<0.001) total SFA over the 4-week period (average decrease of 3.40 g/100 g FA, or 4.9%). When comparing the mean of treatments within week 4, there was no difference (*P*>0.05) between EL, CPLO and MR. For total MUFA there was an overall diet effect (*P*=0.022; Figure 1d). Again, the CPO diet did not change MUFA between weeks 0 and 4 (*P*=0.986) but EL, CPLO and MR all increased (*P*<0.001) total MUFA (average increase at week 4 of 2.38 g/100 g FA, or 8.8%), with no difference (*P*>0.05) between total MUFA means for EL, CPLO and MR at week 4. Diet affected (*P*<0.001) total PUFA concentration (Figure 1e), with EL, CPLO and MR all increasing (*P*<0.05) PUFA over the 4-week period. At week 4 total PUFA concentration was highest (*P*<0.01) in farms feeding EL, with the concentration for CPLO and MR farms being similar (*P*=0.899).

## Discussion

Feeding linseed and rapeseed oil supplements is an effective strategy for decreasing milk fat SFA and increasing unsaturated FA concentrations (Glasser *et al*., 2008). However, effectiveness depends on the oil content of the oilseed, and the amount consumed by the cow. Studies have shown that consuming large amounts of crushed rapeseed results in a substantial decrease in SFA concentration, with a concomitant increase in unsaturated FA (e.g. Kliem *et al*., 2011). Even at more modest levels (i.e. <500 g oilseed oil/cow per day), supplementation with ground rapeseed and ground linseed decreased milk total SFA concentration (Collomb *et al*., 2004; Egger *et al*., 2007; Chen *et al*., 2008). However, it is still unknown whether lower levels, despite being more sustainable at a commercial farm level, would influence bulk milk FA profile for an entire herd. This knowledge is critical for the development of feeding strategies that will affect the entire milk pool, and therefore the milk and dairy product-consuming public.

In the present studies, the target oil intake was 350 g/cow per day. This was the maximum amount of supplement practical to feed taking into account the oil content of each supplement. Most studies investigating effects of oilseeds on milk FA profile have utilised a higher intake (e.g. >500 g oil/cow per day) of oil (Glasser *et al*., 2008), but caution must be exerted at high inclusion levels due to the negative effect on the rumen function and cow performance (Lock and Shingfield, 2004). Increasing the amount fed in the present studies to for example 1 kg oil/cow per day would have meant feeding a total of 1.2 kg CPO, 4 kg EL, 2 kg CPLO and 3.3 kg MR supplements. Feeding incremental levels of MR up to 1250 g oil/cow per day (5.5 kg MR/day) had no effect on DMI and milk yield and composition over a 28-day period (Kliem *et al*., 2011), but an earlier study involving feeding 1214 g oil/cow per day as whole cracked rapeseed decreased DMI, milk yield and milk fat yield (Givens *et al*., 2003). As the present study objective was to investigate levels of supplementation that are practical to feed taking into account the oil content of each supplement, it was not feasible to supplement at higher levels.

Feeding 350 g/oil equivalent of all lipid supplements had no effect on DMI, milk yield or milk composition when compared with a control diet containing no lipid supplement, in agreement with previous studies whereby cows were supplemented with oilseeds supplying similar amounts of oil (Collomb *et al*., 2004; Oeffner *et al*., 2013). This is particularly important when developing a sustainable strategy for use on commercial dairy farms. Increasing the energy density of the diet (e.g. by feeding supplemental oilseeds) can sometimes decrease DMI, which can decrease milk yield (Chilliard *et al*., 2009), but this response varies with oilseed form and amount fed. Supplemental oilseeds can also decrease milk fat and protein concentration. ELs can depress milk fat synthesis when included in a diet higher in NDF content (420 to 430 g/kg DM, Gonthier *et al*., 2005; 308 g/kg DM, Chilliard *et al*., 2009; 397 g/kg DM, Ferlay *et al*., 2010) but have little effect when included in low NDF-containing diets (174 g/kg DM, Oeffner *et al*., 2013), which may reflect the effect of linseed oil on fibre fermentation in the rumen. Experiment 1 in the current study involved TMR diets containing ~330 g/kg DM NDF. However, at the level of oil fed a negative impact on milk fat secretion was unlikely. Experiment 2 resulted in a decreased fat concentration in bulk milk from EL farms after 4 weeks. This may reflect the basal diets fed, as this group fed the highest average ratio of grass silage : maize silage, but diets were not analysed for chemical composition, and detailed dietary information was unfortunately unavailable for some farms in this group. Earlier studies including MR in dairy cow diets reported no effect on milk fat and protein concentrations, at intake levels of up to 1345 g oil/cow per day (Givens *et al*., 2009; Kliem *et al*., 2011).

The lack of difference between EL and Control/CPO in terms of total SFA concentration in experiment 1 appeared to be due to enhanced 18:0 concentration despite decreased 16:0. The other linseed-containing supplement (CPLO) exerted a greater effect on total SFA, mainly due to the numerical decrease in SFA⩽14:0, which is in agreement with an earlier study involving supplementation of calcium salts of linseed oil (Brzóska, 2006). A similar effect in SFA⩽14:0 was observed in the present study when comparing Control with CPO. Intake of total FA from CPLO was greater than that of EL, which suggests that a greater quantity of longer chain PUFA escaped rumen metabolism and inhibited mammary synthesis (via inhibition of acetyl CoA carboxylase activity) of shorter chain SFA (Barber *et al*., 1997). The MR treatment appeared to only affect 14:0 and 16:0 concentration when compared with both Control and CPO, which is in agreement with earlier studies (Givens *et al*., 2009; Kliem *et al*., 2011). In contrast to experiment 1, experiment 2 saw all three oilseed supplements decrease total SFA over a 4-week period, when compared with CPO. The disparity between studies may be due to differences in the basal diets of farms enrolled in experiment 2, which were not under experimental control, or indeed average lactation stage of cows consuming supplements, which varied depending on farm due to differences in calving pattern. The interaction between oilseed supplementation and basal diet has been reported previously (Sterk *et al*., 2011), and a lower rumen pH (e.g. for high starch diets) affects a shift in rumen biohydrogenation pathways so that biohydrogenation of dietary MUFA/PUFA is less complete (Palmquist *et al*., 2005). The average forage : concentrate ratio across all farms (excluding three who were unable to provide this information) was 64 : 36, so overall a greater proportion of forage than in experiment 1. In addition, although the aim during experiment 2 was that each cow consumed 350 g/day additional oil, in reality this may not have occurred. Stage of lactation will affect DMI, and farms involved in experiment 2 were a mixture of all-year-round- and autumn-calving herds, meaning that, perhaps for some cows, all of the supplement was not consumed. Conversely, depending on feeding system, some cows may have had the opportunity to consume more than 350 g/day oil. This highlights the importance of conducting trials on commercial farms to verify experimental results.

The greatest milk fat concentration of *cis*-MUFA was observed for the CPLO diet, which reflected milk fat *cis*-9 18:1 concentration. This was probably derived from both *cis*-9 18:1 intake, and via increased rumen outflow of 18:0 that was subsequently desaturated by mammary ∆9 desaturase, following biohydrogenation of dietary PUFA (for CPLO the daily intake of 18:2 n-6+18:3 n-3 was the greatest PUFA intake across all diets). Supplementation with the linseed-based supplements also increased *cis*-12 18:1 and *cis*-16 18:1 concentrations, which tend to be elevated following linseed supplementation (Lerch *et al*., 2012b) and are biohydrogenation intermediates of 18:3 n-3 (Shingfield *et al*., 2010). Increases in the concentration of individual *trans*-18:1 isomers following CPLO supplementation reflect the higher intake of PUFA. *Trans*-6-8 and *trans*-9 18:1 concentrations were greater in milk from CPLO and MR-fed cows than those supplemented with EL. This may be due to the increased intake of *cis*-9 18:1 on the CPLO and MR diets, as *trans*-7 and *trans*-9 18:1 largely originate from isomerisation of *cis*-9 18:1 in the rumen (Mosley *et al*., 2002). All three oilseed diets increased *trans*-12 18:1, which is an intermediate of *cis*-9 18:1, 18:2 n-6 and 18:3 n-3 biohydrogenation (Shingfield *et al*., 2010). There was no difference (*P*>0.1) in milk fat total MUFA concentration between the CPO diet and those containing oilseed supplements. However, in experiment 2 the same supplements increased MUFA concentration after 4 weeks compared with CPO, when fed at the same rate. Again, this could be related to basal diet and/or consumption rate. Increasing the proportion of concentrates in the diet (from 60 : 40 to 40 : 60 forage : concentrate) resulted in a greater increase in total MUFA following EL supplementation (Neveu *et al*., 2013).

EL appeared to increase the concentration of most *trans*-18:2 isomers to a greater extent than the other supplements. A proportion of *cis*-9, *trans*-13 18:2 is synthesised endogenously by the action of mammary ∆9 desaturase on *trans* 18:1 (Shingfield *et al*., 2008), and *trans*-11, *cis*-15 18:2 is an intermediate of rumen 18:3 n-3 metabolism (Shingfield *et al*., 2010). Supplementing cow diets with 350 g oil as CPLO resulted in the greatest increase in total *trans* FA compared with both the Control and the CPO diet. Supplementing with similar quantities of rapeseed oil (Halmemies-Beauchet-Filleau *et al*., 2011; Jacobs *et al*., 2011) and linseed oil (Jacobs *et al*., 2011) resulted in much greater concentrations of total *trans* FA.

The effectiveness of an oilseed supplement for increasing concentrations of milk PUFA depends on the PUFA content of the supplement, as well as its physical form. Transfer efficiency of 18:3 n-3 (daily yield of 18:3 n-3 from milk as a percentage of 18:3 n-3 intake) following EL supplementation is generally greater compared with supplementing as linseed oil (Chilliard *et al*., 2009), perhaps due to a degree of rumen protection by the EL matrix. In experiment 1, 18:3 n-3 transfer efficiency due to EL was greater (*P*<0.001) than that of CPLO (6.3% *v*. 4.6%, respectively). This, coupled with the greater concentration in milk fat *trans* FA following the CPLO diet, suggests that the rumen inertness of PUFA in CPLO was lower. However, there was no difference (*P*=0.721) between EL and CPLO in terms of transfer efficiency of 18:2 n-6. Rumen stability of calcium soaps of FA is assumed to be inversely related to the degree of FA unsaturation (Chouinard *et al*., 1998), so it is possible that 18:2 n-6 within the CPLO diet may have experienced partial rumen protection.

The objective of the present experiments was to demonstrate whether inclusion of oilseed supplements in dairy cow diets at a commercially viable level could have meaningful impact on the FA profile of milk, in terms of decreasing SFA and increasing *cis*-MUFA and -PUFA. Results from experiment 1 suggest SFA can be decreased by an average of between 1.8 (compared with CPO) and 3.5 (compared with control diet) g/100 g FA. However, compared with CPO, oilseed supplements decreased bulk milk SFA concentration by on average 3.4 g/100 g in the commercial study. A decrease of this magnitude if applied to average UK winter (October to February) milk with a SFA concentration of 71.3 g/100 g FA (Kliem *et al*., 2013) would have small impact on UK adult SFA consumption if applied to all dairy products consumed (change from 13.4% to 13.2% and 12.9% to 12.7% food energy intake for men and women, respectively; intake data derived from Bates *et al*., 2014). Nonetheless it would be a move in the right direction, and would remove SFA from the food chain whilst replacing them with MUFA and PUFA, albeit with the inclusion of small proportions of *trans* FA. At current intake levels ruminant-derived *trans* FA are thought not to have negative effects on human health (Mozaffarian, 2006) although further research is required to inform on the isomer-specific effects of ruminant-derived *trans* FA (Gebauer *et al*., 2011).

In conclusion, results from these two experiments were in broad agreement, in that inclusion of modest amounts of different oilseed-based supplements in dairy cow diets decreased milk SFA concentration by replacement with unsaturated FA. This effect can be observed in a change-over study involving five cows, and also a continuous study involving 3081 cows, and thus demonstrates successful transfer of scientific principle to commercial practice.
